# Exploring Cancer Incidence Trends by Age and Sex Among 14.14 Million Individuals in China From 2007 to 2021: Population-Based Study

**DOI:** 10.2196/55657

**Published:** 2024-08-07

**Authors:** Yingchao Yuan, Chang Liu, Moning Guo, Zhong Xin, Guanjie Chen, Yue Yang, Jianpeng Zheng, Bai Zang, Jinkui Yang

**Affiliations:** 1 Beijing Key Laboratory of Diabetes Research and Care, Department of Endocrinology and Metabolism, Beijing Diabetes Institute Beijing Tongren Hospital Capital Medical University Beijing China; 2 Beijing Municipal Health Big Data and Policy Research Center Beijing China; 3 The Center for Research on Genomics and Global Health National Human Genome Research Institute National Institutes of Health Bethesda, MD United States

**Keywords:** cancer, incidence, trend, sex-based, women

## Abstract

**Background:**

Sex is a crucial factor in the development, progression, and treatment of cancer, making it vital to examine cancer incidence trends by sex for effective prevention strategies.

**Objective:**

This study aimed to assess the incidence of cancer in China between 2007 and 2021, with a focus on sex-based trends.

**Methods:**

A population-based cancer registry comprising 14.14 million individuals was maintained between 2007 and 2021 by the Beijing Municipal Health Big Data and Policy Research Center. The age-standardized rates (ASRs) of cancers were calculated using the Segi population. The average annual percentage of change (AAPC) was evaluated using the joinpoint regression model, while the Bayesian age-period-cohort model was used to predict cancer incidence in the next 10 years.

**Results:**

From 2007 to 2021, the study included 651,342 incident patients with cancer, of whom 51.2% (n=333,577) were women. The incidence indicated by the ASR for all cancers combined was 200.8 per 100,000 for women and 184.4 per 100,000 for men. The increase in incidence indicated by AAPC for all malignancies combined significantly increased in women between 2007 and 2021 (AAPC=3.1%; *P*<.001), whereas it remained constant in men (AAPC=0.3%; *P*=.30). Although the overall incidence of all cancers indicated by AAPC increased in young men (AAPC=3.2%; *P*=.01), the greatest increase was observed among young women (AAPC=6.1%; *P*<.001). The incidence rate ratio for cancer in women increased among subsequent younger generations compared with patients born in the 1962-1966 cohort. The ASR in women will increase 1.6-fold over the next 10 years, with women having twice the incidence rate of men by 2031.

**Conclusions:**

The rising incidence of cancer among women in China has become a growing concern, emphasizing the need for increased efforts in cancer prevention and early screening, especially among young women.

## Introduction

Cancer is a major cause of death worldwide, imposing a significant economic burden on public health systems [1,2]. Cancer incidence varies by location and is influenced by many variables, such as sex, ecology, environment, population, culture, and genetics [3,4]. China, which is undergoing rapid economic and social development, is experiencing a shift in its cancer spectrum, facing a significant cancer burden resembling patterns seen in higher-income countries [5,6]. Despite various organizations reporting cancer statistics in China [7,8], few reports on cancer incidence trends in recent years are available, especially limited population–based cancer registration reports. Understanding the incidence of cancer and its trends over time is essential to guide local and global cancer control efforts.

Since biological sex is a genetic modulator of illness pathogenesis and response to medical treatment, sex differences are a focus of current clinical practice and medical research [9]. Previous research has noted a higher incidence of certain cancers in men than women, possibly attributed to factors like tobacco, alcohol, and occupational exposures [10]. As China undergoes industrialization, more women are joining fields that were formerly dominated by men, impacting their lifestyle, reproductive choices, and health care practices [11]. Analyzing differences in cancer risks between sexes is crucial due to variations in carcinogenicity and susceptibility to risk factors. However, trends in cancer incidence between sexes in China, especially in recent years, remain unclear.

Epidemiological studies often prioritize older people [7,8], yet scant attention is paid to age-specific cancer incidence trends. Analyzing cancer incidence in young people is crucial for understanding environmental and lifestyle impacts on cancer onset and formulating effective prevention policies [12].

This study used population-based cancer registry data to analyze the trends in cancer incidence among men and women in different age groups from 2007 to 2021, with a particular focus on young individuals. Additionally, we aimed to forecast the future burden of cancer for both sexes in each age group. Our objective was to reflect the recent real trends in cancer incidence, with a specific emphasis on sex differences, to provide insights for future research and assist policy makers in implementing appropriate cancer control measures.

## Methods

### Data Sources

This survey was conducted among the population located in the greater Beijing area, which comprises 16 districts, including 8 urban areas and 8 suburbs, situated in northeast China. The data used in this study were obtained from the Beijing Municipal Health Big Data and Policy Research Center, which is responsible for the health of the population in Beijing.

The cancer data used in this study were obtained from all 207 medical centers (Table S1 in Multimedia Appendix 1) that were certified to diagnose cancer. These centers are required by the Beijing Municipal Health Big Data and Policy Research Center to report the diagnoses and information of all cancer patients for registration, research, and publication of government work reports to evaluate the impact of new policies and adjust prevention strategies for cancer [13]. To ensure the quality of medical record data, the Beijing Municipal Health Big Data and Policy Research Center conducts a supervisory inspection visit every year and invites experts to check the accuracy of the diagnoses [14]. The population data, classified by 5-year age group and sex, were obtained from the statistics of the Beijing Municipal Bureau of Statistics [15].

The numerator of the estimated incidence includes all newly diagnosed cancer patients registered in Beijing who were followed from January 1, 2007, or the registration date if later than that, to December 31, 2021, and the denominator of the estimated incidence was the registered population of all ages in Beijing during this period. The registered population increased from 12.12 million in 2007 to 14.14 million in 2021. We compared the cancer data uploaded by the Beijing Municipal Health Big Data and Policy Research Center for patients with cancer before 2007 and removed those who had already developed cancer before 2007 to ensure that only new cancer cases from 2007 were included. The flow chart of the cancer registry is shown in Figure S1 in Multimedia Appendix 2.

Case ascertainment was subjected to quality control based on the criteria of the Chinese Cancer Registration and International Agency for Research on Cancer/International Association of Cancer Registries (IARC/IACR). Cancer types were classified according to the *International Classification of Diseases, 10th Revision* (*ICD-10*) and *International Classification of Disease for Oncology, 3rd Edition* (*ICD-O-3*) [7], and were divided into various categories. Benign tumors, in situ cancers, and secondary cancers were excluded from the analysis. The specific cancer categories are shown in Table S2 in Multimedia Appendix 1.

### Ethical Considerations

The study was based solely on deidentified aggregate data, without access to individual records. This study protocol was approved by the Ethics Committee of Beijing Tongren Hospital, Capital Medical University (TREC2023-KY006). Every procedure was carried out in compliance with the applicable rules and regulations. Furthermore, the Ethics Committee of Beijing Tongren Hospital, Capital Medical University, waived the requirement for informed consent due to the study including no secondary data or personal information.

### Statistical Analysis

We calculated incidence rates by dividing the number of new cancer cases by the number of observed persons per year by age group and sex. The age-standardized rates (ASRs) of cancers were calculated using the Segi population [7]. Time trends in cancer incidence from 2007 to 2021 were expressed as average annual percentage of change (AAPC), and the *Z* test was used to assess whether the AAPC was statistically different from zero. We used the terms “increase” or “decrease” when AAPC was statistically significant (*P*<.05). When there was no statistical difference in AAPC, we used the term “stable”. We used Joinpoint (version 4.8.0.1; National Cancer Institute [NCI]) software to calculate the AAPC of cancer [16]. To reduce the likelihood of reporting spurious changes in trends during this period, all models were limited to a maximum of 2 joinpoints (3-line segments). To assess the incidence trends of cancers across age groups, we divided the population into those younger and older than 50 years.

To estimate net cohort effects on the incidence of cancers, an age-period-cohort model was used to fit each cancer type through the analysis tool provided on the NCI website [17,18]. In the model, incidence rate ratio (IRR) was appropriately divided into continuous 5-year birth cohort groups (1922-1926 [1924 cohort], 1927-1931 [1929 cohort],...1992-1996 [1994 cohort], 1997-2001 [1999 cohort]). We chose the 1964 cohort (1962-1966) as the referent birth cohort because it is midway between examined cohorts.

We used the Bayesian age-period-cohort (BAPC) model to predict the incidence of cancers by 2031 [19]. The model was used to fit cancer incidence in the 5-year age group for each calendar year from 2007 to 2021. The BAPC model uses integrated nested Laplace approximations (INLA) for full Bayesian inference and generates age-specific and age-standardized projected rates. When interest lies in the predictive distribution, Poisson noise is automatically added [20]. For statistical analysis, we used the *BAPC* package (version 0.0.36) in R Studio (version 1.1.383; RStudio Inc) and Joinpoint. We considered *P*<.05 to be statistically significant.

## Results

### Cancer Incidence Rates in China: 2007 to 2021

**Figure 1:**
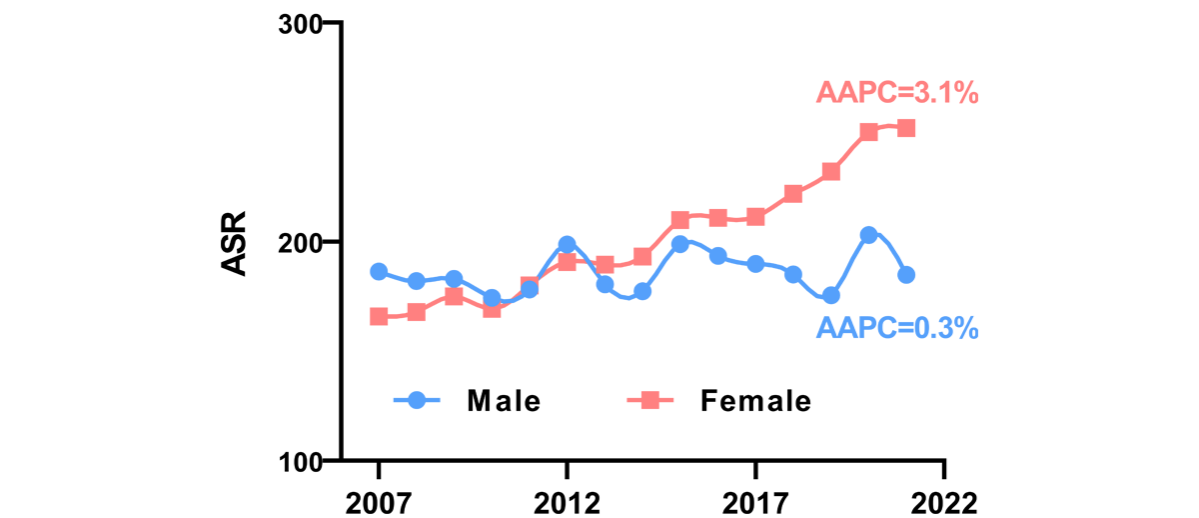
Age-standardized incidence rates (ASRs) and incidence trends for all cancers combined by sex, 2007 to 2021. The average annual percentage of change (AAPC) was 3.1% (*P*<.001) in women and 0.3% (*P*=.30) in men.

We identified a total of 651,342 cancer patients in the greater Beijing area from January 1, 2007, to December 31, 2021, including 317,765 men and 333,577 women. The top 20 malignancies by incidence in the Chinese population from 2007 to 2021 are listed in Table 1. The ASR by the Segi population for overall cancer was higher in women than in men, with rates of 200.8 per 100,000 for women compared to 184.4 per 100,000 for men (Figure 1 and Table 1). To better reflect the situation of the Chinese population, we standardized on the Chinese population census in 2000. The age-standardized incidence rate for the Chinese standard population for all cancers in men was 180.4 per 100,000, and in women it was 216.0 per 100,000 (Table 1).

Lung cancer was the most prevalent cancer occurring in men, with a rate of 69.1/100,000, accounting for 21.6% (68,602/317,765) of all new cancer cases. For women, breast cancer was the most prevalent type of cancer, with a rate of 78.9/100,000, making up 23.4% (78,157/333,577) of all new cases (Table 1). Lung cancer incidence in women has risen dramatically in the last 6 years, surpassing that of men in 2021. Additionally, thyroid cancer incidence in women consistently remained higher than in men (Figure S2 in Multimedia Appendix 2).

**Table 1:** Incidence rates for all cancers combined and the 20 most common cancers stratified by sex.

Sites	Male					Female			
	Cases^a^, n (proportion, %)	Incidence, 1/100,000	ASR^b,c^, 1/100,000	ASR^d^, 1/100,000	Cases^a^, n (proportion, %)		Incidence, 1/100,000	ASR^b,c^, 1/100,000	ASR^d^, 1/100,000
All sites	317,765 (100)	320.2	184.4	180.4	333,577 (100)		337.1	200.8	216.0
Head	6533 (2.1)	6.6	4.5	4.0	3928 (1.2)		4.0	2.8	2.6
Esophagus	12,108 (3.8)	12.2	6.3	6.0	2703 (0.8)		2.7	1.2	1.3
Stomach	22,278 (7)	22.5	11.5	11.2	9905 (3)		10.0	5.2	5.6
Colon	23,021 (7.2)	23.2	11.8	11.4	18,702 (5.6)		18.9	8.9	9.6
Rectum	22,570 (7.1)	22.7	12.0	11.6	14,360 (4.3)		14.5	7.2	7.6
Liver	23,649 (7.4)	23.8	13.9	13.8	7301 (2.2)		7.4	4.0	4.0
Biliary tract	4555 (1.4)	4.6	2.3	2.2	3108 (0.9)		3.1	1.4	1.5
Pancreas	6727 (2.1)	6.8	3.5	3.3	5454 (1.6)		5.5	2.6	2.8
Lung	68,602 (21.6)	69.1	36.0	34.6	48,992 (14.7)		49.5	25.4	26.7
Breast	—^e^	—	—	—	78,157 (23.4)		78.9	47.1	51.2
Cervix	—	—	—	—	9288 (2.8)		9.4	6.3	7.2
Uterus	—	—	—	—	17,943 (5.4)		18.1	10.6	11.0
Ovary	—	—	—	—	11,226 (3.4)		11.3	7.1	7.6
Prostate	19,062 (6)	19.2	8.8	8.3	—		—	—	—
Kidney	12,422 (3.9)	12.5	7.6	7.5	5990 (1.8)		6.1	3.7	3.7
Bladder	17,540 (5.5)	17.7	8.7	8.2	5606 (1.7)		5.7	2.6	2.8
Nervous system	7847 (2.5)	7.9	7.9	8.0	5864 (1.8)		5.9	5.9	5.9
Thyroid	14,282 (4.5)	14.4	10.7	13.1	38,384 (11.5)		38.8	28.4	34.1
Lymphoma	7653 (2.4)	7.7	5.4	5.3	6060 (1.8)		6.1	3.9	4.1
Leukemia	7434 (2.3)	7.5	7.7	7.0	5339 (1.6)		5.4	5.6	5.0
All other sites and unspecified	41,482 (13.1)	41.8	26.0	25.0	35,267 (10.6)		35.7	21.2	21.1

^a^The number of cases is the sum of the number of cases of cancers for 15 years (2007 to 2021) of data.

^b^ASR: age-standardized incidence rate.

^c^ASR for all cancers based on the Segi population.

^d^ASR for all cancers based on the 2000 Chinese census population.

^e^Not applicable.

### Trends in Cancer Incidence by Sex

The ASR by the Segi population for all malignancies combined considerably rose in women between 2007 and 2021 (AAPC=3.1%; *P*<.001), whereas it remained constant in men (AAPC=0.3%; *P*=.30) (Figure 1). Additionally, there was a significant increase in the overall cancer ASR by the Chinese standard population in women (AAPC=3.3%; *P*<.001), while the overall incidence rate in men remained stable (AAPC= 0.5%; *P*=.50) (Table S3 in Multimedia Appendix 1).

Among the top 20 cancers, the largest increase in ASR by the Segi population for women was seen in thyroid cancer (AAPC=16.2%; *P*<.001), followed by lung cancer (AAPC=6.7%; *P*<.001). Although the ASR by the Segi population for breast cancer rose by 2.6% per year, breast cancer still accounted for nearly a quarter of all cancers in women, warranting more attention (Figure S2 in Multimedia Appendix 2). The incidence of cancer among women continued to rise despite the removal of lung cancer, breast cancer, or thyroid cancer, respectively, but remained stable when all 3 cancers were removed at once, indicating that breast, thyroid, and lung cancer are the primary causes of the rise in the incidence of cancer among women (Table S4 in Multimedia Appendix 1). Cancer incidence in women also increased for colon (1.3% per year), rectal (1%), and uterine (3.8%) cancers. However, the ASR by the Segi population for women revealed a substantial decline in esophageal, gastric, liver, biliary, pancreatic, cervical, and ovarian cancers (Figure S2 in Multimedia Appendix 2).

Among the 20 most common cancers in men, ASR by the Segi population notably rose for colon (2.9% annually), rectal (2.7%), prostate (2.5%), kidney (1.7%), and thyroid (17.6%) cancers. Conversely, esophageal, gastric, liver, and pancreatic cancers saw declines (AAPC was –6.2%, –4%, –6.9%, and –3%, respectively; all *P*<.001), while others remained stable (Figure S2 in Multimedia Appendix 2). Trends in cancer incidence in the Chinese standard population mirrored those in the Segi population (Table S3 in Multimedia Appendix 1).

Moreover, considering that female-specific cancers account for a larger proportion of cancers in women, we compared cancer rates in women by excluding sex-specific cancers, subtracting prostate cancer from male cancers and breast, cervical, ovarian, and uterine cancers from female cancers. Despite stable male cancer rates (AAPC=0.2%; *P*=.50), female cancer rates increased significantly (AAPC=3.7%; *P*<.001), surpassing male rates in 2021 (191.0/100,000 for women vs 176.2/100,000 for men) (Table S5 and S6 in Multimedia Appendix 1).

### Trends in Cancer Incidence by Age Group

**Figure 2:**
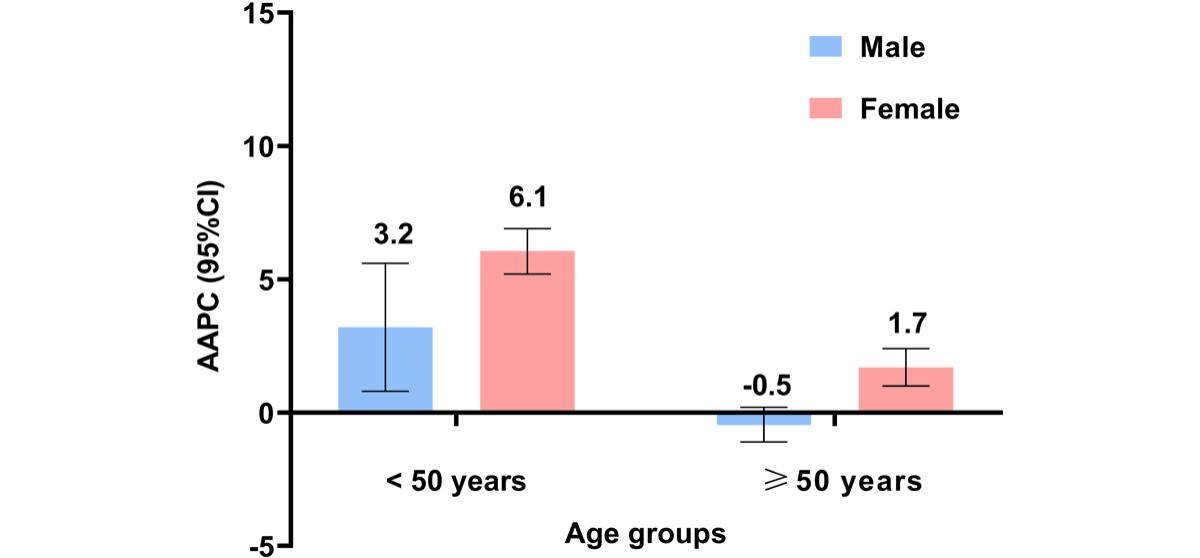
Average annual percentage change (AAPC) for all cancers combined by sex and age. The AAPC was 3.2% (*P*=.01), 6.1% (*P*<.001), –0.5% (*P*=.15), and 1.7% (*P*<.001) for younger men and women and older men and women, respectively.

The overall cancer incidence in women increased significantly between 2007 and 2021, with a more substantial increase observed in younger women (aged <50 years) than older women (aged ≥50 years) (AAPC=6.1%; *P*<.001 vs 1.7%; *P*<.001, respectively). In contrast, the ASR remained stable for men overall (AAPC=0.3%; *P*=.30), but increased considerably in younger men (AAPC=3.2%; *P*=.01) and tended to stabilize in older men (AAPC=–0.5%; *P*=.15). The greatest increase in cancer incidence was observed among young women (Figure 2).

**Figure 3:**
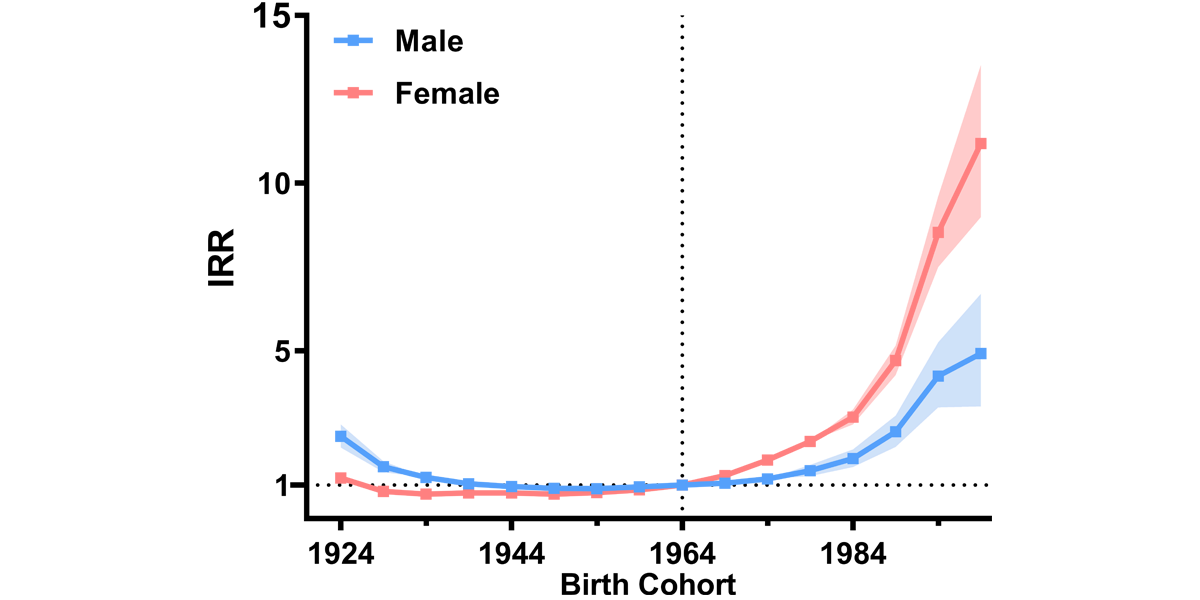
Incidence rate ratio (IRR) by birth cohort for all cancers by sex, 2007-2021.

Likewise, the incidence rate ratio (IRR) by birth cohort for all cancers showed a progressively increasing trend in younger generations, especially among young women. For instance, the IRR for all cancers in women rose to 1.75 (95% CI 1.67-1.82) for those born in the 1974 cohort and 11.02 (95% CI 8.99-13.53) for those born in the 1999 cohort compared to those born in the 1964 cohort. In contrast, the IRR for men only increased to 1.18 (95% CI 1.09-1.29) for those born in the 1974 cohort and 4.73 (95% CI 3.34-6.70) for those born in the 1999 cohort (Figure 3).

In young adults, lung cancer incidence rose in both sexes, notably more in women (13.5% vs 3.5% in men). Among those older than 50 years, lung cancer continued increasing in women (4.6%) but stabilized in men. Head and colon cancer incidence surged faster in young women than young men, and yet more in older men than older women. Thyroid and kidney cancers were more common in young men, a trend that reversed in older people. Breast and uterine cancers increased across all ages, more so in younger women (breast: 3.7% for young vs 2% for older women; uterus: 4.7% for young vs 3.7% for older women). Prostate cancer showed a similar trend in men (13.9% vs 2.3%). Cervical and ovarian cancer incidences declined, with cervical cancer mainly decreasing among young women (–2.2%) and ovarian cancer decreasing among older women (–2.4%) (Figure S3 in Multimedia Appendix 2).

### Predicted Incidence of All Cancers by Sex and Age

**Figure 4:**
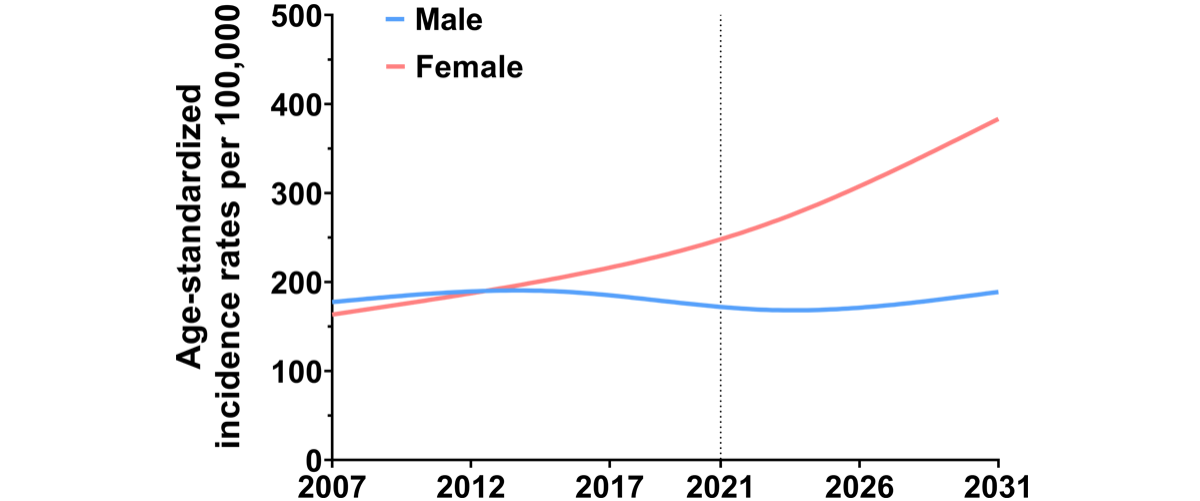
Observed and predicted incidence rate for all cancers by sex, 2007-2021.

Figure 4 shows the observed and predicted ASR for all cancers by sex and age groups from 2007 to 2031. By 2031, the ASR for all cancers combined in women is expected to reach approximately 391.8 cases per 100,000, which is a 1.6-fold increase over the incidence in 2021. For men, the ASR is predicted to be around 199.0 cases per 100,000 people in 2031. This means that the incidence in women is expected to be around twice that of men by 2031.

Over the next 10 years, the incidence of all cancers in women aged 20-74 years is projected to increase, particularly in those younger than 50 years (20-24: 10% increase; 25-29: 19% increase; 30-34: 75% increase; 35-39: 120% increase; 40-44: 115% increase; and 45-49: 64% increase). In men, the incidence is expected to rise in the 25-59 age group, mostly among young adults (25-29: 10% increase; 30-34 years: 48% increase; 35-39: 78% increase; 40-44: 78% increase; 45-49: 57% increase). However, the increase in women is expected to be larger than that in men in the same age group (Figure S4 in Multimedia Appendix 2).

## Discussion

### Principal Findings

This study comprehensively describes the trends in cancer incidence by sex for all age groups from 2007 to 2021 and predicts their future cancer incidence for the next decade. Our analysis reveals that the age-standardized cancer incidence among women has surpassed that of men, with a notable rise in cancer incidence among younger generations. We predict an increasing burden of cancer in the future.

From 2007 to 2021, the sex gap in cancer incidence has been narrowing, with women surpassing men in 2013 and continuing to rise. This shift in incidence is due to changing patterns of cancer incidence among men and women. Like in Western nations, the ASR for all cancers combined significantly rose in women during this period, while it remained unchanged in men [21,22]. Meanwhile, we also observed a concerning trend: the incidence of cancer in individuals younger than 50 years was increasing rapidly, with a rate of 6.1% per year for young women and 3.2% for young men. Therefore, our research fills a gap, as there has been a lack of recent reports on cancer data in the Chinese population, and provides valuable insights into the sex disparities of cancer burden in China.

Among all cancers in women, lung, thyroid, and breast cancers were the main contributors to the rise in incidence. The incidence trend of lung cancer has shifted significantly, accounting for a significant portion of the disparity in overall incidence between men and women. From 2007 to 2021, the incidence of lung cancer in men remained steady, whereas in women, it climbed 6.7% per year and surpassed that of men in 2021. Lung cancer incidence rose in all age groups for women, especially in young women, with an increase of 13.5% each year, while it only increased by 3.5% for young men and remained stable in older men. Similar changes in incidence patterns have also been observed globally in many countries, and the incidence of lung cancer in women is projected to continue to increase significantly through 2035 [23,24].

Changes in smoking habits have led to varying trends in lung cancer rates among men and women. Smoking is a major risk factor for lung cancer, with reports indicating increased smoking rates among Chinese women and youths over the past two decades, while rates declined among men [25]. The rise in lung cancer among women may also be linked to changes in secondhand smoke exposure, especially in major cities like Beijing, Shanghai, and Chengdu, due to crowded living conditions and lax enforcement of smoke-free laws [26]. Studies show that women have a higher lung cancer prevalence compared to men of the same age, despite similar cigarette use levels [27]. Further research should monitor sex differences in lung cancer risk and investigate causes behind the higher incidence in women.

The incidence of breast cancer in women increased by 2.6% per year during our study period, with a greater increase among young women (3.7%), contributing to the overall rise in cancer incidence. Family planning and lifestyle changes in China have led to younger ages at menarche, delayed childbirth, and reduced breastfeeding, among other factors, all of which contribute to an increased prevalence of breast cancer in younger women [28,29]. Occupational risk factors, such as night shift work in a standing posture, have also been linked to breast cancer incidence in Beijing [30].

Our study revealed an upward trend in the incidence of thyroid and prostate cancers, particularly among young individuals. This increase may be partially attributed to the rapid development of monitoring techniques such as ultrasound, computed tomography, magnetic resonance imaging, and needle biopsy, that have significantly increased the detection rate of cancer [31]. However, mounting evidence suggests that there may be a real increase in incidence as well. Inappropriate iodine consumption (either too much or too little) and excessive radiation exposure may contribute to the high prevalence of thyroid cancer, while obesity and a westernized lifestyle have been linked to prostate cancer [32,33].

We observed a distinctive pattern in the incidence trend of gastrointestinal malignancies in this population. Colorectal cancer incidence was on the rise, while esophageal, stomach, and liver cancer incidence gradually declined. This phenomenon can be explained by the distinct risk factors associated with different types of cancer. Chronic infections, particularly *Helicobacter pylori* or hepatitis B and C virus (HBV and HCV), are the predominant risk factors for esophageal, gastric, or liver cancers. However, colorectal cancer is more strongly associated with changes in lifestyle, such as a high consumption of red or processed meats, sedentary behavior, and obesity [34,35].

In recent years, significant advancements in cancer screening in China, driven by government efforts [36], have expanded screening programs. Four large-scale initiatives launched since 2005 now cover 231 county-level areas in 31 provinces, focusing on various cancers [37]. These efforts aim to detect cancer early and raise public awareness of disease prevention. Endoscopy is now the primary method for esophageal cancer screening, dating back to the 1970s. Efforts since 2005 have led to a decline in esophageal cancer incidence [38,39]. Similarly, *H pylori* infection prevalence has decreased due to increased screening, diagnosis, and treatment. Combined with effective endoscopic screening, gastric cancer incidence has significantly decreased [37]. Liver cancer is primarily caused by chronic HBV and HCV infections, aflatoxin exposure, alcohol consumption, and smoking [40,41]. Since 1992, China has implemented systematic hepatitis B vaccination for infants, leading to a notable decline in hepatitis B surface antigen prevalence [42]. Measures to prevent HBV and HCV infections, such as regulating blood products and purifying contaminated water sources, have effectively lowered liver cancer incidence [43]. Cervical cancer incidence has decreased due to widespread screening. A national project launched in 2009 provided free cervical examinations to 11.69 million women aged 35-59 years from 221 counties over 3 years [37,44]. Human papillomavirus (HPV) testing and cytology have increased lesion detection rates and reduced cervical cancer incidence [45]. The introduction of the HPV vaccine in 2017 is expected to further prevent cervical cancer as its availability increases [46,47].

To tackle the rising cancer burden, we propose intensified screening of high-risk populations, especially young women, to improve early detection. Simple, cost-effective screening methods for early symptoms in young and middle-aged populations can also raise awareness of disease prevention. This includes routine thyroid palpation, educating young obese women about breast self-examinations, and exploring tumor biomarkers [48]. Due to the increase in lung cancer among women, efforts should prioritize smoking cessation and reducing secondhand smoke exposure. Government campaigns promoting healthy living are crucial in combating obesity-related cancers like colorectal and breast cancers [49]. Additionally, integrating the HPV vaccine into the national immunization schedule can further reduce cervical cancer rates. Developing an HCV vaccine and interventions to prevent HBV and HCV transmission are ongoing priorities.

### Limitations

Our study benefits from the use of licensed medical practitioners for cancer diagnosis and the latest data covering a population of 14.14 million from 2007 to 2021, allowing us to accurately identify trends in cancer incidence across age groups in China. However, a limitation of our study is that it used data from hospitalized cases, which may underestimate actual cancer morbidity due to financial and medical restrictions and missed diagnoses among patients who did not receive further hospitalization. Additionally, we only reported aggregated incidence; we did not analyze differences in cancer incidence between tissue types and regional, racial, or ethnic subgroups. Future research should consider these factors for a more comprehensive understanding of cancer incidence in China.

### Conclusion

In summary, the disparity in cancer incidence between men and women has been narrowing as a result of economic development and industrialization, and cancer incidence in women, especially young women, has sharply increased in China. With the aging of the young population, the number of new cancer cases among Chinese women is projected to rise rapidly in the future. It is crucial for future cancer prevention to focus on sex variations in cancer risk and conduct research on the etiology of cancer in men and women in order to comprehend and identify potential risk factors. Cancer researchers need to broaden their emphasis on women to elucidate the risks associated with gynecologic cancers. The Chinese government should establish comprehensive policies including targeted prevention, early detection, and treatment programs in the future to control the increasing cancer burden.

## Appendix

Multimedia Appendix 1 Supplementary tables for cancer data in China from 2007 to 2021.

Multimedia Appendix 2 Supplementary figures for cancer data in China from 2007 to 2021.

## Abbreviations

AAPC: average annual percentage of change
ASR: age-standardized rate
BAPC: Bayesian age-period-cohort
HBV: hepatitis B virus
HCV: hepatitis C virus
HPV: human papillomavirus
IARC/IACR: International Agency for Research on Cancer/International Association of Cancer Registries
ICD-10: International Classification of Diseases, 10th Revision
ICD-O-3: International Classification of Disease for Oncology 3rd Edition
INLA: integrated nested Laplace approximations
IRR: incidence rate ratio
NCI: National Cancer Institute

## Data Availability

The data that support the findings of this study are available from the corresponding author upon reasonable request.
