# Supplementary material for: Exploring Cancer Incidence Trends by Age and Sex Among 14.14 Million Individuals in China From 2007 to 2021: Population-Based Study
Source: JMIR Public Health Surveill. 2024 Aug 7;10:e55657. doi: 10.2196/55657 (PMC11339572; doi:10.2196/55657)
Supplement: Multimedia Appendix 1 [file publichealth_v10i1e55657_app1.docx]

**Multimedia Appendix 1. The supplementary tables for cancer data in China from 2007 to 2021**

**Table S1. Hospitals Included (n=207)**

| 1. Peking University Third Hospital 2. Peking University First Hospital 3. Peking University Stomatological Hospital 4. Peking University People's Hospital 5. Peking University Shougang Hospital 6. Peking University Hospital 7. Beijing Fengtai Hospital 8. Beijing Aerospace General Hospital 9. Beijing Huaxin Hospital (The First Affiliated Hospital of Tsinghua University) 10. Beijing Huairou Hospital 11. Beijing Jishuitan Hospital 12. Beijing Jingmei Group General Hospital 13. Beijing Geriatric Hospital 14. Beijing Qinghe Hospital 15. Beijing Bo’ai Hospital 16. Maternal and Child Health Hospital of Changping District, Beijing 17. Nankou Railway Hospital, Changping District, Beijing 18. Nankou Hospital of Changping District, Beijing (Nankou Hospital of Integrated Traditional Chinese and Western Medicine, Changping District, Beijing) 19. Shahe Hospital, Changping District, Beijing 20. Beijing Changping District Hospital 21. Beijing Changping District Integrated Traditional Chinese and Western Medicine Hospital 22. Beijing Changping District Traditional Chinese Medicine Hospital 23. Maternal and Child Health Hospital of Chaoyang District, Beijing 24. Beijing Chaoyang District Hospital of Traditional Chinese Medicine 25. Beijing Chaobaihe Orthopedics Hospital 26. Beijing Weeping Willow Hospital 27. Beijing Daxing District People's Hospital 28. Beijing Daxing District Hospital of Integrated Traditional Chinese and Western Medicine 29. Beijing Sixth Hospital 30. Beijing First Hospital of Integrated Traditional Chinese and Western Medicine 31. The First People's Hospital of Dongcheng District, Beijing 32. The First Hospital of Fangshan District, Beijing 33. Maternal and Child Health Hospital of Fangshan District, Beijing 34. Liangxiang Hospital, Fangshan District, Beijing 35. Beijing Fangshan Hospital of Traditional Chinese Medicine (Fangshan Hospital of Beijing University of Traditional Chinese Medicine) 36. Beijing Fengsheng Traditional Chinese Medicine Orthopedics Specialty Hospital 37. Nanyuan Hospital, Fengtai District, Beijing 38. Tieying Hospital, Fengtai District, Beijing 39. Beijing Fengtai Hospital of Integrated Traditional Chinese and Western Medicine 40. Beijing Anorectal Hospital 41. Beijing Gulou Hospital of Traditional Chinese Medicine 42. Maternal and Child Health Hospital of Haidian District, Beijing 43. Beijing Haidian Hospital 44. Beijing Hepingli Hospital 45. Beijing Red Cross Emergency Rescue Center (Beijing Red Cross Trauma Hospital) 46. Beijing Institute of Occupational Disease Prevention and Control (Beijing Institute of Occupational Disease Prevention and Control) 47. Maternal and Child Health Hospital of Huairou District, Beijing 48. Traditional Chinese Medicine Hospital of Huairou District, Beijing 49. Beijing Muslim Hospital 50. Central Hospital of Beijing Prison Administration Bureau 51. Beijing Jiangong Hospital 52. Beijing Geriatrics Hospital 53. Beijing Longfu Hospital (Beijing Dongcheng Geriatrics Hospital) 54. Beijing Mentougou District Maternal and Child Health and Family Planning Service Center (Beijing Mentougou District Maternal and Child Health Hospital) 55. Longquan Hospital, Mentougou District, Beijing 56. Beijing Mentougou District Hospital 57. Beijing Mentougou District Traditional Chinese Medicine Hospital (Beijing Mentougou District Geriatrics Hospital) 58. Maternal and Child Health Hospital of Miyun District, Beijing 59. Miyun District Hospital, Beijing 60. Beijing Miyun District Traditional Chinese Medicine Hospital 61. Beijing Pinggu District Hospital 62. Beijing Pinggu District Hospital of Traditional Chinese Medicine 63. Beijing Puren Hospital 64. Beijing Renhe Hospital 65. Beijing Social Welfare Hospital 66. Beijing Shijingshan Hospital 67. Beijing Shunyi District Maternal and Child Health Hospital (Beijing Children’s Hospital Shunyi Women’s and Children’s Hospital) 68. Airport Hospital of Shunyi District, Beijing 69. Beijing Shunyi District Hospital 70. Beijing Shunyi District Chinese Medicine Hospital (Beijing Chinese Medicine Hospital Shunyi Hospital) 71. Maternal and Child Health Hospital of Tongzhou District, Beijing 72. Beijing Tongzhou District Integrated Traditional Chinese and Western Medicine Hospital 73. Beijing Tongzhou District Hospital of Traditional Chinese Medicine 74. Guangwai Hospital of Xicheng District, Beijing (Guangwai Hospital of Xicheng District, Beijing) 75. Beijing Xicheng District Safe Hospital 76. Exhibition Road Hospital, Xicheng District, Beijing 77. Beijing Xuanwu Hospital of Traditional Chinese Medicine 78. Maternal and Child Health Hospital of Yanqing District, Beijing 79. Beijing Yanqing District Hospital (Peking University Third Hospital Yanqing Hospital) 80. Beijing Yangfangdian Hospital 81. Beijing Zhongguancun Hospital (Zhongguancun Hospital of Chinese Academy of Sciences) 82. Beijing Hospital of Integrated Traditional Chinese and Western Medicine 83. Beijing Capital International Airport Hospital 84. Beijing Water Conservancy Hospital 85. Central Hospital of Beijing Railway Branch 86. Beijing Xiaotangshan Hospital 87. Beijing Yanhua Hospital 88. Beijing Hospital 89. The Third Affiliated Hospital of Beijing University of Chinese Medicine 90. Dongfang Hospital, Beijing University of Chinese Medicine 91. Dongzhimen Hospital, Beijing University of Chinese Medicine 92. Huguosi Hospital of Traditional Chinese Medicine Affiliated to Beijing University of Chinese Medicine 93. Beijing Traditional Chinese Medicine Hospital Yanqing Hospital (Beijing Yanqing District Traditional Chinese Medicine Hospital) 94. Beijing Cancer Hospital 95. State Grid Corporation Beijing Electric Power Hospital 96. Aviation General Hospital 97. Aerospace Center Hospital 98. Civil Aviation General Hospital 99. Tsinghua University Hospital 100. Yuquan Hospital of Tsinghua University 101. Children's Hospital Affiliated to Capital Institute of Pediatrics 102. Beijing Anzhen Hospital, Capital Medical University 103. Beijing Chaoyang Hospital, Capital Medical University 104. Beijing Ditan Hospital, Capital Medical University 105. Beijing Children's Hospital, Capital Medical University 106. Beijing Obstetrics and Gynecology Hospital, Capital Medical University 107. Beijing Stomatological Hospital, Capital Medical University 108. Beijing Luhe Hospital, Capital Medical University 109. Beijing Shijitan Hospital, Capital Medical University (Beijing Railway General Hospital) 110. Beijing Tiantan Hospital, Capital Medical University 111. Beijing Tongren Hospital, Capital Medical University 112. Beijing Chest Hospital, Capital Medical University 113. Beijing Friendship Hospital, Capital Medical University 114. Beijing Youan Hospital, Capital Medical University 115. Beijing Traditional Chinese Medicine Hospital Affiliated to Capital Medical University 116. Fuxing Hospital Affiliated to Capital Medical University 117. Xuanwu Hospital, Capital Medical University 118. Emergency Management Department Emergency General Hospital (formerly Coal General Hospital) 119. Beijing Tibetan Hospital, China Tibetology Research Center 120. China Aerospace Science and Industry Corporation 731 Hospital 121. China Nuclear Industry Beijing 401 Hospital 122. Peking Union Medical College Hospital, Chinese Academy of Medical Sciences 123. Fuwai Hospital, Chinese Academy of Medical Sciences 124. Plastic Surgery Hospital of Chinese Academy of Medical Sciences 125. Chinese Academy of Medical Sciences Cancer Hospital 126. Guang'anmen Hospital, China Academy of Chinese Medical Sciences 127. Wangjing Hospital of China Academy of Chinese Medical Sciences 128. Xiyuan Hospital, China Academy of Chinese Medical Sciences 129. Eye Hospital of China Academy of Chinese Medical Sciences 130. China-Japan Friendship Hospital 131. Beijing Second Hospital 132. Xinhua Hospital, Tongzhou District, Beijing 133. Maternal and Child Health Hospital of Daxing District, Beijing 134. Xinkang Hospital, Daxing District, Beijing 135. Maternal and Child Health and Family Planning Service Center of Fengtai District, Beijing 136. The Second People's Hospital of Miyun County 137. Beijing Shijingshan Hospital of Traditional Chinese Medicine 138. Beijing Rehabilitation Hospital Affiliated to Capital Medical University 139. Sanbo Brain Hospital, Capital Medical University 140. Beijing Shangdi Hospital 141. Beijing Xicheng District Maternal and Child Health Family Planning Service Center (Beijing Xicheng District Maternal and Child Health Hospital) 142. The Second Hospital of Huairou District, Beijing 143. Maternal and Child Health Hospital of Pinggu District, Beijing 144. Peking University International Hospital 145. Beijing Delcony Orthopedic Hospital 146. Beijing Meizhong Yihe Women's and Children's Hospital 147. Beijing Southern Suburb Cancer Hospital 148. Huanxing Cancer Hospital, Chaoyang District, Beijing 149. Shuangqiao Hospital, Chaoyang District, Beijing 150. The First Maternity and Child Health Hospital of Dongcheng District, Beijing 151. Beijing Pinggu Yuexie Hospital 152. Beijing Sijiqing Hospital 153. Rehabilitation Hospital Affiliated to National Research Center for Rehabilitation Assistive Devices 154. Beijing Boren Hospital 155. Beijing Chaoyang Emergency Rescue Center 156. Beijing Fengtai Youanmen Hospital 157. Beijing Harmony Women's and Children's Hospital 158. Beijing United Family Hospital 159. Beijing Jingcheng Boai Rehabilitation Hospital 160. Beijing Mary Women and Children's Hospital 161. Beijing Tsinghua Chang Gung Memorial Hospital 162. The Second Maternal and Child Health Hospital of Dongcheng District, Beijing 163. Beijing Wuzhou Women and Children's Hospital 164. Beijing New Milestone Cancer Hospital 165. Beijing Asian Games Village Meizhong Yihe Women's and Children's Hospital 166. Beijing Tongrentang Hospital of Traditional Chinese Medicine 167. Beijing Wangfu Hospital of Integrated Traditional Chinese and Western Medicine 168. Beijing Huangcheng Femoral Head Necrosis Specialist Hospital 169. Beijing Tongji Oriental Integrated Traditional Chinese and Western Medicine Hospital 170. Beijing Lianke Chinese Medicine Nephropathy Hospital 171. Beijing David Hospital of Traditional Chinese Medicine 172. Beijing Dongyuan Hospital of Traditional Chinese Medicine 173. Beijing Fengtai InBev Hospital of Integrated Traditional Chinese and Western Medicine 174. Beijing Hou Liping Rheumatism Hospital of Traditional Chinese Medicine 175. Beijing Huajun Hospital of Traditional Chinese Medicine 176. Beijing Kangyide Pulmonary Hospital of Integrated Traditional Chinese and Western Medicine 177. Beijing Yao Medical Hospital 178. Beijing Huatan Hospital of Integrated Traditional Chinese and Western Medicine 179. Beijing Hui'an Hospital of Integrated Traditional Chinese and Western Medicine 180. Beijing Yuhe Integrated Traditional Chinese and Western Medicine Rehabilitation Hospital 181. Beijing Chaoyang Integrated Traditional Chinese and Western Medicine Emergency Rescue Center 182. Beijing Fengtai Medical Star Integrated Traditional Chinese and Western Medicine Hospital 183. Beijing Sihui Traditional Chinese Medicine Hospital 184. Beijing Weida Traditional Chinese Medicine Cancer Hospital 185. Beijing Annabelle Obstetrics and Gynecology Hospital 186. Beijing Baiziwan Hemei Women's and Children's Hospital 187. Beijing Changfeng Hospital 188. Beijing Fengtai Guangji Integrated Traditional Chinese and Western Medicine Hospital 189. Beijing Fengtai Kangtai Hospital 190. Beijing Fengtai Sanlu Ju Hospital of Integrated Traditional Chinese and Western Medicine 191. Beijing Jingke Gantai Hospital 192. Beijing 618 Factory Hospital 193. Beijing Mairui Orthopedic Hospital 194. Beijing Red Cross Peace Orthopedic Hospital 195. Beijing Timber Factory Staff Hospital 196. Beijing Sanhuan Yinghe Hospital 197. Beijing Wanliu Meizhong Yihe Women's and Children's Hospital 198. Beijing New Century Women's and Children's Hospital 199. Beijing Peking University Medical Rehabilitation Hospital 200. Beijing Fengtai Huashan Hospital 201. Beijing Jingdu Children's Hospital 202. Beijing Lu Daopei Hospital 203. Beijing Eurasian Cancer Hospital 204. Beijing Fengtai District Elderly Association Lianhuachi Rehabilitation Hospital 205. Beijing Taihe Obstetrics and Gynecology Hospital 206. Beijing New Century Children's Hospital 207. Beijing Youlian Eye, Ear, Nose and Throat Hospital |
| --- |

| **Table S2. ICD-10 code for the 20 most common cancers** | |
| --- | --- |
| Site | ICD-10 |
| Head | C00-10, C12-14 |
| Esophagus | C15 |
| Stomach | C16 |
| Colon | C18 |
| Rectum | C19-21 |
| Liver | C22 |
| Biliary tract | C23 |
| Pancreas | C25 |
| Lung | C33-34 |
| Breast | C50 |
| Cervix | C53 |
| Uterus | C54-55 |
| Ovary | C56 |
| Prostate | C61 |
| Kidney | C64 |
| Bladder | C67 |
| Nervous system | C47, C70-72 |
| Thyroid | C73 |
| Lymphoma | C81-85, C88, C96 |
| Leukemia | C91-95 |
| All other sites and unspecified | A-O |

| **Table S3. Trends in age-standardized incidence rates by Chinese standard population for the 20 most common cancers by sex, 2007 to 2021** | | |
| --- | --- | --- |
| Sites | AAPC | *P*-Value |
|  | 2007-2021 |  |
| All Sites | **2.1*** | <0.001 |
| **Male** |  |  |
| All sites | 0.5 | 0.5 |
| Head | -0.5 | 0.765 |
| Esophagus | **-6.1*** | <0.001 |
| Stomach | **-3.9*** | <0.001 |
| Colon | **2.8*** | 0.004 |
| Rectum | **2.6*** | <0.001 |
| Liver | **-7.0*** | <0.001 |
| Biliary tract | -0.6 | 0.324 |
| Pancreas | **-3.0*** | 0.006 |
| Lung | 1.2 | 0.301 |
| Breast | / |  |
| Cervix | / |  |
| Uterus | / |  |
| Ovary | / |  |
| Prostate | **2.7*** | <0.001 |
| Kidney | **1.9*** | 0.003 |
| Bladder | -0.4 | 0.48 |
| Nervous system | -1.3 | 0.66 |
| Thyroid | **18.5*** | <0.001 |
| Lymphoma | -0.3 | 0.685 |
| Leukemia | **-3.4*** | 0.036 |
| **Female** |  |  |
| All sites | **3.3*** | <0.001 |
| Head | -0.3 | 0.841 |
| Esophagus | **-9.3*** | <0.001 |
| Stomach | **-3.2*** | <0.001 |
| Colon | **1.3*** | <0.001 |
| Rectum | 0.9 | 0.054 |
| Liver | **-7.1*** | <0.001 |
| Biliary tract | **-2.5*** | 0.016 |
| Pancreas | **-3.8*** | <0.001 |
| Lung | **6.8*** | <0.001 |
| Breast | **2.6*** | <0.001 |
| Cervix | **-1.7*** | <0.001 |
| Uterus | **3.9*** | <0.001 |
| Ovary | **-1.7*** | 0.001 |
| Prostate | / | / |
| Kidney | **1.4*** | 0.02 |
| Bladder | **-1.8*** | 0.001 |
| Nervous system | -0.2 | 0.949 |
| Thyroid | **16.3*** | <0.001 |
| Lymphoma | -0.2 | 0.666 |
| Leukemia | **-2.4*** | 0.044 |

| **Table S4. Trends in age-standardized incidence rates by Segi’s population for different cancer sites by sex, 2007 to 2021^a^** | | | |
| --- | --- | --- | --- |
| cancer site | AAPC | | |
|  | All ages | ＜50 years | ≥50 years |
| All male cancers | 0.3 | **3.2*** | -0.5 |
| All female cancers | **3.1*** | **6.1*** | **1.7*** |
|  |  |  |  |
| All male cancers | 0.3 | **3.1*** | -0.5 |
| All female cancers except for breast cancer | **3.3*** | **7.3*** | **1.5*** |
|  |  |  |  |
| All male cancers except for lung cancer | 0.3 | **4.2*** | **-0.6*** |
| All female cancers except for lung cancer | **2.7*** | **5.7*** | **0.7*** |
|  |  |  |  |
| All male cancers except for thyroid cancer | -0.7 | 0.2 | -0.4 |
| All female cancers except for thyroid cancer | **1.3*** | **3.1*** | **1.3*** |
|  |  |  |  |
| All male cancers except for lung and thyroid cancer | -0.9 | -0.5 | **-0.8*** |
| All female cancers except for breast,lung and thyroid cancer | -0.5 | 0.6 | **-0.8*** |
| ^a^The AAPC is signifigcantly different from zero (*P*＜.05) |  |  |  |

| **Table S5. Different cancer incidence rates stratified by sex, 2007 to 2021a** | | | | |
| --- | --- | --- | --- | --- |
| Year | All male cancers | All female cancers | All male cancers except for prostate cancer | All female cancers except for breast, cervix, uterus and ovary cancer |
|  | ASR | ASR | ASR | ASR |
|  | 1/100000 | 1/100000 | 1/100000 | 1/100000 |
| 2007 | 186.5 | 166.0 | 174.8 | 108.7 |
| 2008 | 182.0 | 168.0 | 167.8 | 112.2 |
| 2009 | 183.1 | 175.1 | 167.8 | 112.6 |
| 2010 | 174.4 | 169.4 | 160.7 | 111.7 |
| 2011 | 178.2 | 180.1 | 165.2 | 118.2 |
| 2012 | 198.7 | 190.8 | 181.2 | 129.9 |
| 2013 | 180.6 | 189.6 | 165.1 | 126.5 |
| 2014 | 177.5 | 193.3 | 164.8 | 132.9 |
| 2015 | 199.0 | 209.9 | 181.7 | 147.9 |
| 2016 | 193.7 | 210.9 | 184.0 | 148.5 |
| 2017 | 189.9 | 211.4 | 179.5 | 148.9 |
| 2018 | 185.0 | 221.9 | 174.2 | 155.5 |
| 2019 | 175.5 | 232.0 | 166.6 | 167.2 |
| 2020 | 203.0 | 250.0 | 194.5 | 183.5 |
| 2021 | 184.9 | 252.0 | 176.2 | 191.0 |
| 2007-2021 | 184.4 | 200.8 | 172.1 | 139.0 |
| ^a^ASR, Age-standardized incidence rates are based on the Segi’s population | | | | |

| **Table S6. Trends in age-standardized incidence rates based on the Segi’s population for cancers by sex, 2007 to 2021** | | |
| --- | --- | --- |
| Sites | AAPC | P-Value |
|  | 2007-2021 |  |
| All male cancers | 0.3 | 0.3 |
| All male cancers except for prostate cancer | 0.2 | 0.5 |
| All female cancers | 3.1* | <.001 |
| All female cancers except for breast, cervix, uterus and ovary cancer | 3.7* | <.001 |
