# Supplementary material for: Exploring Cancer Incidence Trends by Age and Sex Among 14.14 Million Individuals in China From 2007 to 2021: Population-Based Study
Source: JMIR Public Health Surveill. 2024 Aug 7;10:e55657. doi: 10.2196/55657 (PMC11339572; doi:10.2196/55657)
Supplement: Multimedia Appendix 2 [file publichealth_v10i1e55657_app2.docx]

**Multimedia Appendix 2. The supplementary figures for cancer data in China from 2007 to 2021**

**Figure S1
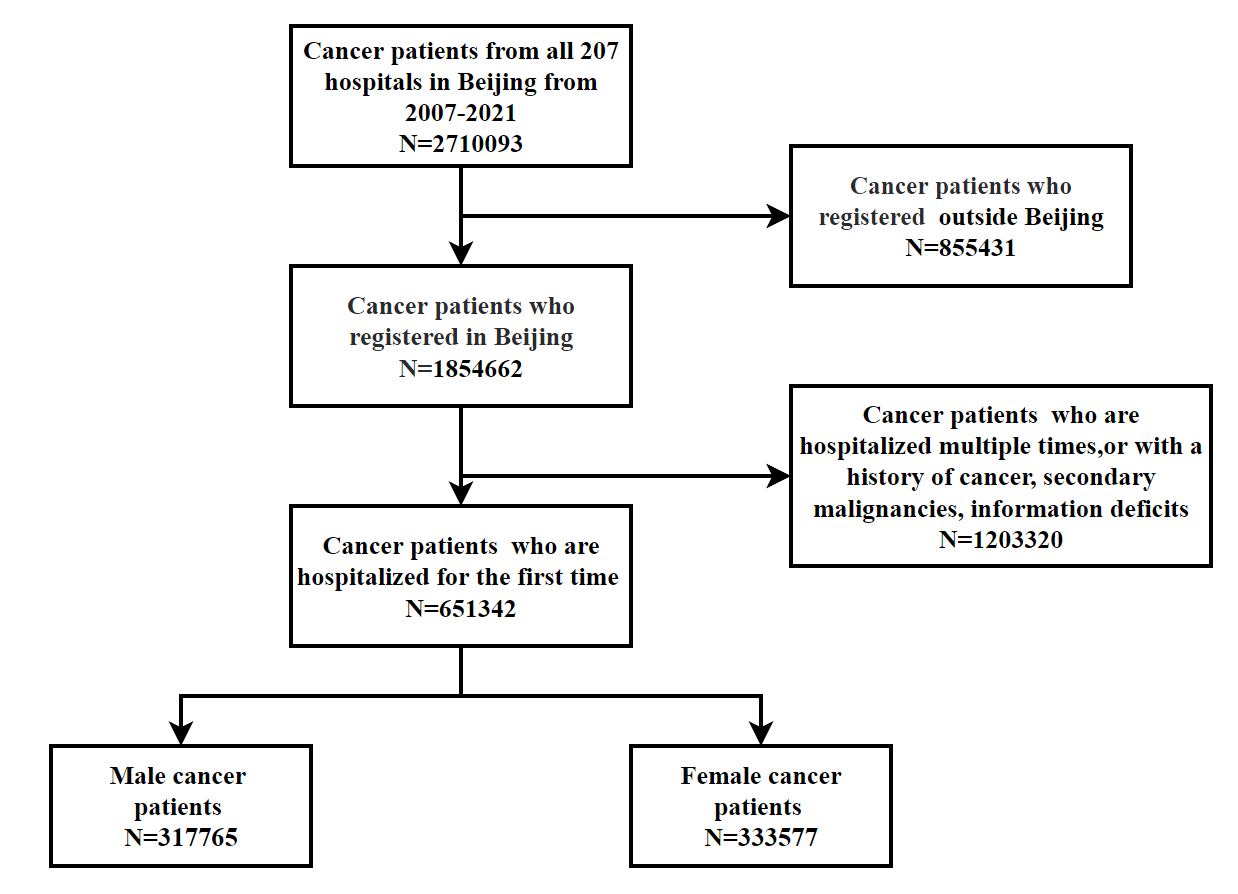
**

Figure S1. The flow chart of the cancer registry.

Figure S2. Age-standardized incidence rates and incidence trends for 20 most common cancers stratified by gender, 2007 to 2021

^a^The AAPC are significantly different from zero (*P*<.05).

Figure S3. Average annual percentage change incidence for 20 most common cancers stratified by gender and ages.

^a^The AAPC are significantly different from zero (*P*<.05).


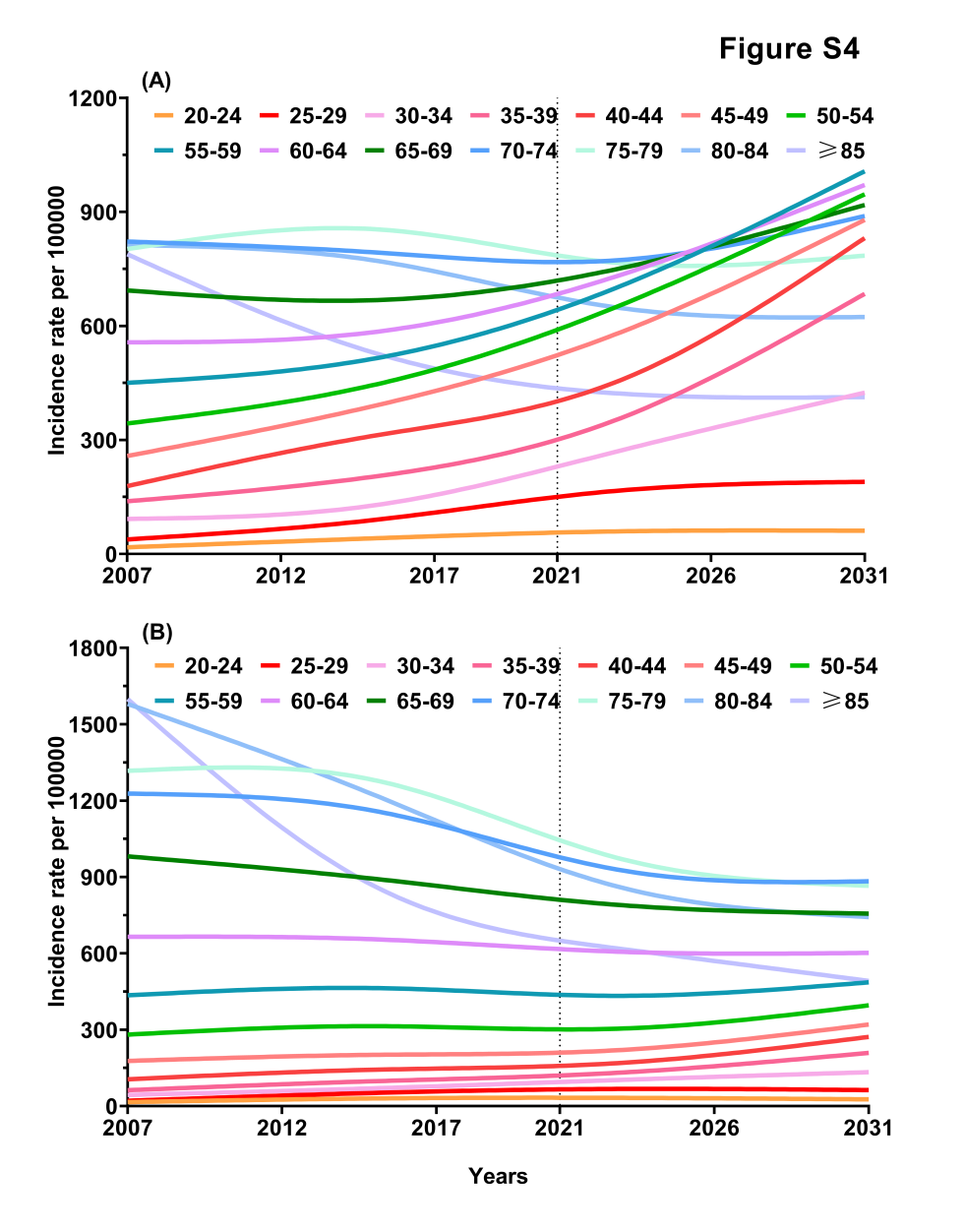


Figure S4. Observed and predicted incidence rate (age-standardized to Sigi Standard Population) for the all cancers from 2007-2031. A: all female cancers by ages; B: all male cancers by ages.
